# The Moderating Role of Resilience in the Relationship Between Occupational Stressors and Psychological Distress Among Aviation Pilots in Pakistan

**DOI:** 10.3390/ejihpe15100206

**Published:** 2025-10-11

**Authors:** Ali Ijaz, Anila Amber Malik, Tayyeba Ahmad, Waqas Hassan, Sofia Mastrokoukou, Claudio Longobardi

**Affiliations:** 1 Department of Psychology, University of Karachi, Karachi 75270, Pakistan; ali.ijazahmed@student.ku.edu.pk (A.I.); aamalik@uok.edu.pk (A.A.M.); 2 Department of Psychology, National University of Modern Languages, Islamabad 38000, Pakistan; tayyeba.ahmad@numl.edu.pk; 3 Department of Psychology, Riphah Institute of Clinical and Professional Psychology, Lahore 54660, Pakistan; waqas.hassan@riphah.edu.pk; 4 Department of Political and Social Sciences, University of Salerno, 84084 Fisciano, Italy; 5 Department of Psychology, University of Turin, 10124 Turin, Italy; claudio.longobardi@unito.it

**Keywords:** resilience, aviation pilots, depression, anxiety, stress, fatigue, coping strategies, mental health

## Abstract

Background: Aviation is one of the most demanding professions, exposing pilots to persistent stressors such as fatigue, irregular schedules, and high safety responsibility. These conditions heighten vulnerability to depression, anxiety, and stress (DAS), yet the protective mechanisms mitigating such effects remain less well understood. Objective: This study examined the roles of resilience, coping strategies, and fatigue in predicting DAS among commercial airline pilots. Method: A sample of 200 pilots completed validated self-report measures: the Connor–Davidson Resilience Scale (CD-RISC), the Coping Inventory for Stressful Situations (CISS), the Fatigue Severity Scale (FSS), and the Depression Anxiety Stress Scale (DASS-21). Data were analyzed using bivariate correlations, hierarchical multiple regression, and mediation/moderation analyses via the PROCESS macro. Results: Resilience was negatively correlated with total DAS scores (r = −0.46, *p* < 0.001), while fatigue (r = 0.42, *p* < 0.001) and avoidance coping (r = 0.38, *p* < 0.001) were positively correlated. The regression model accounted for 46% of the variance in DAS (*R*^2^ = 0.46). Task-focused coping predicted lower stress levels, whereas avoidance coping predicted higher anxiety and depression. Resilience moderated the relationship between stress and depression, buffering the impact of stress on mood outcomes. Mediation analyses indicated that coping styles partially explained the protective effect of resilience. ANOVA results confirmed that pilots with high resilience reported significantly lower depression scores than those with medium or low resilience, *F*(2, 197) = 6.72, *p* < 0.01. Conclusions: Resilience emerged as both a direct and indirect buffer against psychological strain in aviation. These findings underscore the importance of promoting adaptive coping and resilience training, alongside effective fatigue management, to enhance pilot well-being and maintain safety in aviation systems.

## 1. Introduction

Aviation is a high-risk, high-stakes industry, where pilots are expected to perform optimally under constant mental and physical pressure (Cullen et al., 2017; Mendonca et al., 2025). The nature of aviation work, including long flight times, irregular schedules that disrupt circadian rhythms, frequent travel across time zones, and the continuous responsibility for passenger safety, presents pilots with unique psychological and physiological challenges (Wu et al., 2016). Occupational stressors of this type have been consistently linked to impaired wellbeing and elevated symptoms of depression, anxiety, and fatigue (Aljurf et al., 2018). Understanding not only prevalence but also protective factors is therefore essential, as pilot mental health has direct implications for flight safety. These findings underscore the need to identify protective psychological mechanisms—particularly resilience and coping—that sustain pilots’ mental health and operational safety.

### 1.1. Resilience as a Protective Factor

Resilience is a concept defined as the “demonstration of positive adaptation in the face of significant adversity faced by an individual” (Cherng et al., 2022). Rather than being a fixed trait, resilience is conceptualized as a dynamic process that interacts with coping strategies and workplace characteristics (Cahill et al., 2021). In aviation, resilience may act as a personal resource that buffers occupational stressors and reduces vulnerability to psychological distress.

This study focuses on resilience as both a direct protective factor and a moderator of stress–depression associations. Prior work suggests that resilience, alongside adaptive coping, may reduce vulnerability to depression, anxiety, and stress (DAS) in pilots (Cahill et al., 2021). Nevertheless, the mechanisms by which resilience impacts pilots’ mental health have not yet been sufficiently explored. Assessing resilience as part of a bigger framework that includes coping and fatigue mechanisms constitutes a more complicated notion of psychological adjustment in aviation.

### 1.2. Prevalence of Mental Health Symptoms in Pilots

Aviation pilots are exposed to unique occupational stressors that can significantly affect their mental health and overall well-being. Studies have consistently shown the prevalence of mental health problems such as depression, anxiety, and stress among pilots. For example, a study by Wu et al. (2016) reported that 12.6% of commercial airline pilots met criteria for depression, and 4.1% reported suicidal ideation in the previous two weeks. The study also highlighted strong associations between fatigue, sleep problems, and adverse mental health outcomes. Similarly, a study by Aljurf et al. (2018) of commercial airline pilots from the Gulf Cooperation Council found that 34.5% had abnormal depression scores, and 68.3% suffered from severe fatigue. Additional evidence from other aviation professionals confirms this trend. Görlich and Stadelmann (2020) reported that, among a sample of 1100 cabin crew members, clinically relevant symptoms of depression increased from about 8% before COVID-19 to 23% during the pandemic, and stress levels were nearly three times higher than before the pandemic. This evidence reinforces the conceptualization of psychological vulnerability in aviation occupations and shows that chronic fatigue, job insecurity, and disrupted work schedules are central contributors to declines in mental health. Despite growing empirical evidence of distress, much less is known about the personal resources—such as resilience and adaptive coping—that may buffer or mitigate these adverse effects.

### 1.3. Coping Strategies and Performance

Occupational stress is strongly linked to lower job satisfaction and higher depression and anxiety (Xu et al., 2022). In the aviation industry, coping strategies are among the most important contributors to pilots’ emotional adjustment and occupational functioning (Cahill et al., 2021). Research has shown that pilots who use task-based coping, such as problem solving, cognitive reframing, and planful coping, report significantly lower levels of stress and fatigue compared to those who use avoidant or emotional coping strategies (Ozel & Hacioglu, 2021).

Importantly, adaptive coping styles can mitigate these negative effects, whereas maladaptive strategies such as avoidance exacerbate distress (Xu et al., 2022). In line with the broader literature on coping, Carver (1997) identified avoidance and disengagement coping as maladaptive responses associated with poorer emotional outcomes. Pilots who used task-oriented coping, such as problem solving and planning, reported better well-being and performance, while disengagement or avoidance coping was more likely to increase distress and burnout.

Recent evidence further suggests that coping may operate as a dynamic process that translates individual resilience into improved mental health outcomes, reducing the likelihood of fatigue-induced errors and enhancing flight performance (Zhao et al., 2025a, 2025b).

In line with the transactional model of stress and coping (Lazarus & Folkman, 1984), we therefore expect coping strategies to partially mediate the protective effect of resilience, functioning as a key mechanism through which personal resources translate into improved emotional outcomes and operational safety.

### 1.4. Fatigue and Safety Relevance

The level of fatigue is another critical aspect that affects the mental health and performance of pilots. Scientific evidence shows that fatigue is highly prevalent among pilots and is closely associated with stress, sleep disturbances, and mood disorders (Kallus & Blattner, 2025). In regulatory contexts, 70–90% of pilots report moderate to severe fatigue, often in the context of sleep disorders and daytime sleepiness (Reis et al., 2016; Venus, 2022). Risks associated with the fatigue–stress–sleep disturbance triad appear to be more acute for short- and medium-haul pilots (Reis et al., 2016).

Such high levels of fatigue are not harmless: they are recognized as factors that impair decision-making, attention, and reaction times, thereby increasing the likelihood of cockpit errors and compromising flight safety (ICAO, 2023).

Fatigue should be recognized not only as a symptom, but also as a psychological and physiological antecedent of DAS. In theory, chronic fatigue depletes self-regulatory and cognitive resources, reducing the ability to respond to emotional demands and to use adaptive coping strategies (Cahill et al., 2021; Zhao et al., 2025a). This hypothesis aligns with the Job Demand–Resource (JD-R) model (Bakker & Demerouti, 2017), which indicates that chronic exposure to high demands without sufficient recovery contributes to strain and psychological symptoms. In the JD-R model, resilience and coping are conceptualized as personal resources that may buffer the impact of fatigue on mental health. Fatigue serves as a proximal predictor of DAS symptoms, while resilience and coping are hypothesized as moderating and mediating variables, respectively, in the pilot stress-health pathway.

### 1.5. Conceptual Gaps and Present Study

Few studies have examined resilience as a moderator of the stress–depression link in aviation or coping strategies as mediators of resilience’s protective effects (Cahill et al., 2021). Moreover, most evidence remains cross-sectional and descriptive. Although several recent investigations have explored pilot mental health and influencing factors (Ozel & Hacioglu, 2021; Yu et al., 2022), integrated models that include resilience, fatigue, and coping within a unified predictive framework are still scarce.

Most research has examined only individual variables, such as fatigue risk management or burnout prediction, without tracing the psychosocial processes linking stress exposure to mental health outcomes (Cahill et al., 2021; Zhao et al., 2025a).

The current study adopts an integrative approach by combining the JD-R model (Bakker & Demerouti, 2017) with the transactional model of stress and coping (Lazarus & Folkman, 1984). This dual-theoretical perspective enables examination of both mediation and moderation processes, revealing the deeper psychosocial interaction between resilience and coping, and how these collectively contribute to psychological adjustment in pilots.

Specifically, this study extends the literature by (a) examining resilience as a moderating factor that may attenuate the positive association between stress and depression; (b) identifying coping strategies as mediators of resilience’s protective influence; and (c) including fatigue as a concurrent predictor of mental health to link cognitive and physiological components of pilot well-being. To the best of our knowledge, this is one of the first empirical studies conducted outside the North American aviation context; it extends the literature to the South Asian region and focuses on commercial pilots serving in Pakistan.

This perspective enriches contextual understanding of how these mechanisms operate, shifting the literature from prevalence reporting to more process-oriented research. Such research can inform the development of targeted resilience training, coping-based interventions, and organizational policies to promote pilot well-being and in-flight safety as part of operational risk reduction.

### 1.6. Theoretical Framework

The theoretical framework for this research is based on the JD-R model, which assumes that job demands (e.g., stress) can lead to burnout and negative health outcomes, while job resources, including personal resources such as resilience, can mitigate these effects (Bakker & Demerouti, 2017). This model has recently been adapted to aviation psychology to conceptualize how pilots balance operational demands and psychological resources under conditions of fatigue and time pressure (Cahill et al., 2021; Yu et al., 2022). The JD-R framework is particularly relevant to understanding the psychological challenges pilots face and the potential benefits of resilience training and coping strategies in high-stress occupations.

To be more specific, in the JD-R model, Zhao et al. (2025a) incorporated emotion-regulation mechanisms by showing that resilience and coping behaviors replicated the effect of emotional regulation on pilot burnout. Zhao et al. (2025b) also demonstrated that resilience mitigated the relationship between perceived stress and job burnout, and that cognitive reappraisal is a favorable form of emotion regulation in their study.

High-profile incidents, such as the Germanwings crash in 2015, underscore the systemic importance of pilot mental health. Beyond aviation, evidence from other high-risk professions such as policing and healthcare shows similar vulnerability to stress and burnout, yet pilots face unique organizational barriers that heighten the importance of resilience-building interventions (Ozel & Hacioglu, 2021). Despite growing awareness, many pilots continue to suffer in silence because they fear professional consequences. The early identification of psychological stress and the promotion of resilience-building measures are therefore not only matters of individual well-being but also systemic safety priorities. Within this conceptual frame, the present study applies the JD-R model to empirically examine how resilience, coping, and fatigue interact to shape pilots’ mental health outcomes.

### 1.7. Aim of the Study

This study examines the interactive relationships among resilience, coping strategies, and fatigue in relation to DAS in commercial pilots. Consistent with existing literature, we view resilience as a protective factor that may also moderate and reduce the negative impact of stress on depression (Cahill et al., 2021). Similarly, coping strategies are considered mediating processes between resilience and mental health improvement, in line with studies of pilots and other high-risk occupational groups (Xu et al., 2022). This study aims to clarify the pathways connecting resilience, coping, and fatigue, and to provide a foundation for evidence-based psychological and workplace wellbeing interventions in aviation.

The following hypotheses are tested:

**H1.**  *Resilience will be negatively associated with DAS.*

**H2.**  *Fatigue will be positively associated with DAS.*

**H3.**  *Task-focused coping will be associated with lower DAS; avoidance coping with higher DAS.*

**H4.**  *Resilience will moderate the stress–depression relationship.*

These hypotheses were tested using hierarchical multiple regression to predict DAS from resilience, fatigue, and coping strategies, as well as mediation and moderation analyses. To ensure theoretical consistency, this study is primarily based on the JD-R model (Bakker & Demerouti, 2017). According to this framework, job demands such as fatigue and stress are predictors of psychological strain, while personal resources such as resilience and adaptive disposition buffer against these effects. Within this framework, resilience is considered a personal resource that moderates the relationship between occupational stressors and mental health deficits, while coping is seen as a behavioral response that mediates this relationship. This integrated conceptualization combines elements of Masten’s resilience theory and Lazarus and Folkman’s transactional model of stress and coping into a unified construct, providing a comprehensive framework for understanding pilots’ psychological functioning and forming a basis for policy and intervention measures in aviation. Findings from this study aim to inform evidence-based psychological interventions and policy initiatives promoting pilot wellbeing as an operational safety priority.

## 2. Materials and Methods

### 2.1. Participants

The sample for this research consisted of 200 licensed commercial airline pilots (85% male, 15% female) from national and international carriers in Pakistan. Participants were between 25 and 55 years old and had at least two years of flying experience to ensure adequate exposure to the demands of the occupation. The invitation was distributed through the operations departments of six major airlines in Pakistan, two international and four domestic. The survey link was shared internally using official communication channels, including verified corporate email lists, pilot WhatsApp groups, and direct contact with training and human resources departments at the respective airlines. Approximately 400 invitations were sent, resulting in an overall response rate of 49.9%.

Eligibility was ascertained by a screening question confirming whether respondents were currently employed as licensed commercial pilots by a domestic or international airline. The research team also verified pilots’ credentials through the Pakistan Civil Aviation Authority (PCAA) registry. The sample was diverse in age, gender, type of experience, and type of airline, supporting the representativeness of the results. The sample consisted predominantly of mid-career pilots, with coded mean scores indicating moderate to high levels of experience and an average flight time of 70–90 h per month. Work-related characteristics, including years of flight experience, monthly flight hours, and type of flight operation (domestic, international, or both), were recorded and subsequently included as control variables in the regression analyses to account for potential occupational influences. Demographic information is reported in Table 1.

A quantitative research design was used to examine relationships among resilience, fatigue, coping strategies, and psychological outcomes (depression, anxiety, and stress). This design allowed for the systematic testing of hypotheses concerning the interplay between occupational stressors and individual psychological resources, thereby contributing empirical evidence to inform targeted mental health and safety policies in aviation.

### 2.2. Instruments

All instruments used in this study were standardized psychological scales with established reliability and validity in occupational and clinical research. The English versions were administered, as English is the official language of aviation communication in Pakistan.

Connor-Davidson Resilience Scale (CD-RISC)**.** The CD-RISC (Connor & Davidson, 2003) contains 25 items rated on a 5-point Likert scale from 0 (not true at all) to 4 (true nearly all the time). The total score ranges from 0–100, with higher scores reflecting greater resilience. Example items include “I am able to adapt to change” and “I can bounce back after illness or adversity”. The Cronbach’s alpha was good (α = 0.84). Given the established factor structure of the CD-RISC in occupational samples, the present study focused on reliability indices. Formal permission to use the CD-RISC was obtained from the copyright holder, Dr. Jonathan Davidson, prior to data collection, in accordance with the instrument’s licensing requirements.

Coping Inventory for Stressful Situations (CISS). The CISS (Endler & Parker, 1990) distinguishes three coping styles: emotion-oriented, task-oriented, and avoidance-oriented (with distraction and social diversion as subcomponents). This theoretical background supports the relevance of coping styles as partly dispositional, but also sensitive to occupational contexts. Example items include “focus on the problem and see how I can solve it” (task-oriented) and “take time out and escape the situation” (avoidance). The Cronbach’s alpha was good (α = 0.82).

Fatigue Severity Scale (FSS). The FSS (Krupp et al., 1989) is a nine-item questionnaire assessing the severity and functional impact of fatigue on daily life. Items are rated on a 7-point Likert scale, with higher scores indicating greater fatigue. Example items include “Fatigue affects my physical performance” and “Fatigue interferes with my work, family, or social life”. Cronbach’s alpha was 0.84. The FSS has been widely validated and is particularly relevant to aviation, where fatigue is a critical safety issue.

Depression, Anxiety, and Stress Scale (DASS-21). The DASS-21 (Lovibond & Lovibond, 1995) is a 21-item self-report questionnaire, with seven items per subscale. Items are rated on a 4-point scale (0 = did not apply to me at all, 3 = applied to me very much or most of the time). Example items include “I found it difficult to relax” (stress) and “I felt that life was meaningless” (depression). Cronbach’s alphas were good (α = 0.82 for the total scale).

### 2.3. Procedure

The researchers used a cross-sectional design and conducted the study from October to December 2024 using an anonymous online questionnaire created in Google Forms. The instrument was developed in English—the operational language of aviation in Pakistan—and was pilot-tested for clarity and technical precision with eight captains. Minor wording adjustments were made before data collection. Recruitment was carried out through the operations and human resources departments of six airlines in Pakistan (two international and four local). Each department distributed an invitation letter outlining the study’s purpose, procedures for maintaining anonymity, and the estimated completion time of 15–20 min. The survey link was shared across multiple official platforms: (a) corporate email circulars sent to all currently active flight crew; (b) verified WhatsApp groups used for roster communications; and (c) printed flyers posted in crew briefing areas. Approximately 400 licensed pilots received the invitation to participate, and data collection lasted six weeks. Two reminder messages were sent at two-week intervals to improve response rates, which concluded at 49.9%.

Before starting the questionnaire, participants read a detailed information sheet that included the study’s purpose, potential benefits for future pilots, absence of risk, a description of data handling procedures, and their right to withdraw at any time without consequences. Informed consent was obtained electronically by participants selecting a confirmation box. Next, a mandatory screening question required pilots to confirm they held a valid commercial pilot license and at least two years of active flight experience; those who did not meet these criteria were directed to a termination page. The questionnaire consisted of five sections: (1) demographic and professional information, (2) resilience scale, (3) coping strategies inventory, (4) fatigue scale, and (5) the Depression, Anxiety, and Stress Scale (DASS-21). Participants could revisit and revise their answers before submitting the survey. IP logging was disabled, and each device could submit only one response to limit duplicates. All data were encrypted and stored on password-protected secure drives accessible only to the principal investigators. The survey did not include or retain identifiable information. The study was conducted in accordance with the ethical principles stated by the American Psychological Association (APA, 2020) and the 1964 Declaration of Helsinki and its later revisions. Ethical approval was granted by the Institutional Review Board of the College of Karachi, Pakistan (Protocol No. 10(ASS)44, approved 14 October 2024). Data management protocols followed General Data Protection Regulation (GDPR) requirements, and all analyses were completed using anonymized datasets.

### 2.4. Data Analysis

Descriptive statistics and intercorrelations between key variables were first examined. Before main analyses, data were screened for assumptions of normality, linearity, and multicollinearity. Variance inflation factors ranged from 1.25 to 2.17, indicating no multicollinearity. Residuals were approximately normally distributed, and a Breusch–Pagan test suggested mild heteroscedasticity in the depression model; therefore, HC3 robust standard errors were applied.

Work-related variables, including flight hours per month, years of flight experience, and type of flights, were entered as control variables in the first step of the hierarchical regression analyses to account for occupational differences among participants.

Hierarchical multiple regression was used to predict depression, anxiety, and stress from resilience, fatigue, and coping strategies. Each model’s overall fit was evaluated using F, degrees of freedom, *p* values, and *R*^2^.

Moderation analysis tested whether resilience buffered the effect of stress on depression, and mediation analysis examined coping strategies as mediators of the resilience–depression link. Bootstrapping with 10,000 samples (Hayes & Preacher, 2010) was used to estimate confidence intervals. All analyses were two-tailed with *α* = 0.05. Model fit indices (R^2^ for regressions, *η*^2^ for ANOVA) were used to support interpretation. All analyses were conducted using IBM SPSS Statistics (Version 25) and Hayes’ PROCESS macro (Model 1 for moderation and Model 4 for mediation; Hayes, 2013). Cases with incomplete data were excluded using listwise deletion, resulting in minor variation in sample size (N) across some analyses. Missing data were minimal (less than 5%) and appeared randomly distributed across variables. Little’s MCAR test indicated data were missing completely at random (*p* > 0.05).

## 3. Results

Table 2 presents the means, standard deviations, and Pearson correlation coefficients for the main study variables. Results indicated that resilience was significantly negatively correlated with depression (*r* = −0.52, *p* < 0.001), anxiety (*r* = −0.47, *p* < 0.001), and stress (*r* = −0.50, *p* < 0.001). Resilience was also negatively correlated with fatigue severity (*r* = −0.45, *p* < 0.001) and avoidance coping (*r* = −0.31, *p* < 0.01), while showing a positive correlation with task-focused coping (*r* = 0.38, *p* < 0.001). These correlations provide initial support for the hypothesized relationships.

### 3.1. Regression Analyses

Table 3 summarizes the results of the multiple regression analyses. Work-related variables (years of experience, monthly flight hours, and flight type) were entered at Step 1, followed by resilience at Step 2, fatigue at Step 3, and coping strategies at Step 4. The incremental variance explained (Δ*R*^2^) at each step is reported in Table 3.

For depression, the model explained 35% of the variance (*R*^2^ = 0.35, *p* < 0.001). Higher resilience predicted lower depression (*β* = −0.39), while greater fatigue (*β* = 0.31) and use of avoidance coping (*β* = 0.19) were associated with higher depression.

For anxiety, the model accounted for 32% of the variance (*R*^2^ = 0.32, *p* < 0.001). Resilience (*β* = −0.35) and fatigue (*β* = 0.34) were significant predictors, with avoidance coping showing a smaller but positive effect.

For stress, the model accounted for 36% of the variance (*R*^2^ = 0.36, *p* < 0.001). Resilience (*β* = −0.41) and task-focused coping (*β* = −0.13) were linked to lower stress, while fatigue (*β* = 0.29) and avoidance coping (*β* = 0.18) were linked to higher stress.

Together, these models indicate that resilience and coping styles have robust associations with all three mental health outcomes, even after controlling for fatigue. Each hierarchical model demonstrated good overall fit: depression, *F*(7, 192) = 11.76, *p* < 0.001, *R*^2^ = 0.35; anxiety, *F*(7, 192) = 9.62, *p* < 0.001, *R*^2^ = 0.32; and stress, *F*(7, 192) = 7.64, *p* < 0.001, *R*^2^ = 0.36. No multicollinearity was detected (VIF < 2.0), and residual analyses confirmed linearity and homoscedasticity.

### 3.2. Moderation Analysis

**Figure 1:**
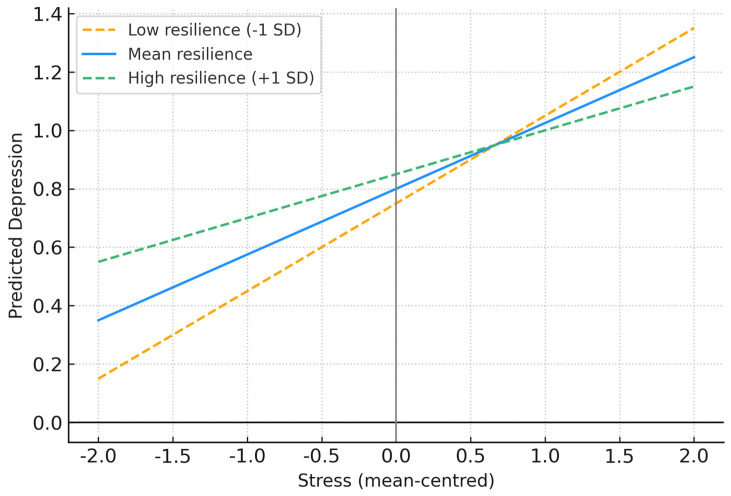
Interaction Between Stress and Resilience in Predicting Depression. Note. The figure illustrates the moderating effect of resilience on the relationship between stress and depression. Simple slopes are plotted at ±1 SD and the mean of resilience. Pilots with high resilience (+1 SD) reported weaker associations between stress and depression compared with those with low resilience (−1 SD). Data analyzed using PROCESS Model 1 (Hayes, 2013); predictors were mean-centered.

Table 4 shows that resilience significantly moderated the relationship between stress and depression (*β* = −0.18, *p* = 0.003). PROCESS Model 1 was used, with mean-centered predictors. As illustrated in Figure 1, pilots with high resilience (+1 *SD*) reported substantially lower depression scores under conditions of high stress compared with those with low resilience (−1 *SD*). This finding confirms H4 and highlights the buffering role of resilience.

### 3.3. Mediation Analysis

Given the stronger theoretical and empirical associations between coping and depressive outcomes, mediation analysis was conducted with depression as the dependent variable and task-focused and avoidance coping as parallel mediators. Emotion-focused coping was excluded due to weak and nonsignificant correlations with depression and resilience.

As shown in Table 5, coping strategies partially mediated the relationship between resilience and depression. PROCESS Model 4 (parallel mediation) was used, with task-focused and avoidance coping specified as separate mediators. Results indicated that resilience positively predicted task-focused coping (*a_1_* path) and negatively predicted avoidance coping (*a_2_* path). In turn, task-focused coping was associated with lower depression (*b_1_* path), whereas avoidance coping was associated with higher depression (*b_2_* path). Both indirect effects (*a_1_b_1_* and *a_2_b_2_*) were significant, as the 95% confidence intervals did not include zero.

### 3.4. Group Comparisons

Table 6 presents the ANOVA results comparing depression levels across three resilience groups (low, medium, and high). A significant main effect was observed, *F*(2, 197) = 18.68, *p* < 0.001, *η*^2^ = 0.16. Tukey’s post hoc comparisons revealed that the high-resilience group reported significantly lower depression scores than both the low- and medium-resilience groups (both *p* < 0.001), while the difference between the low and medium groups was nonsignificant (*p* = 0.07). This categorical analysis complements the regression findings, illustrating that resilience is protective both as a continuous and as a categorical construct.

The results support all four hypotheses. Resilience was consistently protective, fatigue was consistently detrimental, and coping styles exhibited the expected effects. Both the mediation and moderation analyses confirmed that resilience operates through multiple pathways—directly reducing DAS, indirectly via coping strategies, and by buffering the impact of stress on depression. These findings provide a strong empirical foundation for the discussion and its practical implications. Table 2, Table 3, Table 4, Table 5 and Table 6 together offer a comprehensive depiction of these relationships.

## 4. Discussion

The present study examined the relationships between resilience, coping strategies, fatigue, and symptoms of DAS in commercial pilots. The findings support the hypothesised protective role of resilience and adaptive coping, while confirming the detrimental effects of fatigue and avoidance coping. By integrating JD-R model and transactional stress–coping models, this study provides a process-based perspective on pilots’ mental health. It goes beyond merely examining the prevalence of mental health issues by identifying unique psychosocial mechanisms that explain how occupational demands lead to emotional consequences. Below, results are discussed in relation to the four hypotheses.

H1. Resilience and DAS

Resilience was negatively associated with depression, anxiety, and stress, supporting H1. This finding aligns with previous research in military and aviation contexts, which shows that higher resilience buffers psychological strain (Cahill et al., 2021; Li et al., 2023). Importantly, the current study extends this evidence by demonstrating that resilience remains protective even when controlling for fatigue and coping strategies.

However, contrary to previous studies that defined resilience primarily as a trait or selection variable (Cahill et al., 2021), the present findings indicate that resilience is a dynamic process that continues to exert its influence even when controlling for fatigue and coping. The current study’s findings align with emerging evidence in post-pandemic aviation psychology, which suggests that resilience may occur in an oscillatory manner in relation to the balance between perceived organizational support and work demands (Mendonca et al., 2025). Previous research surveying Western pilots found that cognitive flexibility and self-regulatory coping contribute to resilience (Cahill et al., 2021), while Li et al. (2023) identified cognitive flexibility and proactive coping as core resilience components among Chinese pilots. However, the Pakistani pilots in our study appeared to rely more on endurance-based coping and collective efficacy. This suggests that, due to differences in coping orientation -possibly cross-cultural- endurance and collective efficacy may be more resilient than cognitive control, potentially because of their collectivist nature.

This may indicate that resilience is an important psychological resource for pilots, with significant implications for training and selection. The influence of cross-cultural differences in this case provides evidence that resilience-building interventions should be developed through a local conceptual understanding of organizational hierarchies and the occupational cultures of pilots in Eastern domains.

H2. Fatigue and DAS

As predicted, fatigue was strongly associated with higher DAS. This is consistent with earlier studies reporting fatigue as a major contributor to psychological distress and impaired performance among pilots (Rosa et al., 2022). Nevertheless, this study builds on previous work that has measured the impact of fatigue after controlling for resilience and coping, showing that fatigue significantly affects patients’ psychological health—it is not merely a mediator. For example, recent studies (Kallus & Blattner, 2025) have demonstrated this independence, revealing that fatigue continues to predict depressive symptoms even when accounting for sleep quality and hours worked. Furthermore, compared to EASA-based cohorts that have reported fatigue in more than 70% of pilots (Venus, 2022), Pakistani pilots reported moderate and chronic fatigue, which may reflect regional differences in flight schedules and occupational monitoring.

The current findings indicate that fatigue reduces wellbeing and increases the risk of experiencing depression and stress, regardless of an individual’s level of resilience. Thus, managing fatigue risk should be considered a priority for safety and mental health, linking physiological with psychosocial assessments as directed by International Civil Aviation Organization (ICAO) procedure (ICAO, 2023).

H3. Coping strategies and DAS

Task-focused coping was associated with lower DAS, whereas avoidance coping was linked to higher DAS, confirming H3. These results align with the transactional model of stress and coping (Lazarus & Folkman, 1984) and extend earlier aviation research (Xu et al., 2022) in that they distinguish between adaptive and maladaptive coping processes within the same occupational context. This distinction further demonstrates that coping quality rather than quantity produces psychological outcomes. The current findings also support Carver’s (1997) self-regulation theory suggesting that avoidance coping reflects behavioral disengagement, which may sustain rather than resolve stress. In addition, these results emphasize the applied importance of increasing cognitive and behavioral coping flexibility in flight training. Integrating structured interventions that emphasize task-focused coping, such as simulation-based stress inoculation and mindfulness-based attention control methods and cognitive reframing modules, may enhance pilots’ capacity for emotion regulation under the pressures of operational flying. Lastly, cultural factors may impact coping preferences: in environments characterized by collectivism and hierarchy, avoidance may communicate a professional norm for emotional suppression rather than feelings of personal ineffectiveness, suggesting the need for contextually relevant programs for resilience and self-regulation.

H4. Moderating role of resilience

Resilience moderated the stress–depression association, supporting H4. Specifically, the relationship between stress and depression was weaker for pilots with higher resilience. This is consistent with resilience theory (Masten, 2015), which views resilience as a dynamic capacity enabling positive adaptation under adversity (Mendonca et al., 2024). The moderation effect observed here highlights resilience not only as a direct protective factor but also as a buffer against the impact of stress. This finding has practical implications: resilience training could be implemented as part of fatigue risk management and peer-support programs in aviation.

Our research introduces this evidence into aviation and shows that resilience influences both cognitive (adaptive understanding of the threat) and behavioral (continued engagement with the goal) pathways to reduce emotional costs from occupational stressors. This moderating effect differs somewhat from that reported by Cahill et al., who found that resilience protected against anxiety but not depression. These differences may be partly due to the measurement tools used in each study, as the CD-RISC (Connor & Davidson, 2003) employed here is framed in terms of tenacity and adaptability, which are more relevant to high-demand occupational environments. In practice, resilience building could be incorporated into recurrent simulator training, where adaptive decision making under uncertainty is emphasized, as guided by ICAO competence-based training principles.

### 4.1. Broader Implications

The results have both clinical and operational implications. Clinically, they indicate that enhanced resilience and coping flexibility programs should be integrated into existing pilot health initiatives to improve stress tolerance and emotional regulation. Practical interventions may include structured stress inoculation training, mindfulness-based attention control, and cognitive reframing exercises as components of Crew Resource Management (CRM) and recurrent simulator training.

Operationally, it is important to emphasize organizational mechanisms that can monitor fatigue and promote psychosocial safety simultaneously. For example, the airline industry could expand current Fatigue Risk Management Systems (FRMS) policies to include routine psychological screening and data-driven workload adjustments, in line with ICAO Doc 9966 and EASA (European Aviation Safety Agency) CAT.GEN.MPA.100.

Expanding peer-support networks and ensuring confidential reporting processes for mental wellbeing could further reduce stigma and facilitate early intervention. Incorporating resilience training modules into pilot training programs, along with regulatory oversight of compliance and psychosocial monitoring, could provide dual safeguards by strengthening personnel coping resources and reinforcing organizational safety protocols. By addressing both personal and organizational factors affecting wellbeing, the aviation industry can adopt a more proactive and evidence-based approach to mental health and operational safety, consistent with advances in human factors and safety management research.

Collectively, these findings extend current knowledge and continue to support the theoretical integration of the JD-R model and the transactional model of stress and coping. Resilience can now be recognized as both a personal psychological resource and an organizational safety asset, while fatigue and maladaptive coping strategies are identified as key risk factors likely to worsen negative outcomes in all aspects of pilot wellbeing. These implications offer a direct, practical approach to developing targeted interventions, safety policies, and regulatory strategies aimed at maintaining pilot wellbeing and achieving acceptable performance standards.

### 4.2. Limitations

This study has several limitations that need to be considered. Firstly, the design of the cross-sectional study does not allow conclusions to be drawn about causality. This study found an association between resilience and coping and lower levels of depression, anxiety and stress, but longitudinal studies are needed to answer the questions of whether resilience is a resilience to psychological distress and whether pilots who experience better mental health rate themselves as more resilient.

Secondly, the study only used self-report questionnaires, which can lead to biases such as social desirability or under-reporting of symptoms, which is a known issue in aviation as it has a well-known culture typically associated with stigma around mental health issues. No objective data was available to increase the robustness of the data. For example, actigraphy could provide potential sources of data in relation to fatigue and it would have been useful to obtain performance data through simulator testing.

Thirdly, the participants were all professional pilots, all of whom were based in Pakistan. This means that the results cannot be generalized to all pilot populations working in a different cultural, legal or organizational context. Given the international importance of aviation organizations such as the ICAO, the EASA, and the Federal Aviation Administration (FAA), cross-national comparative studies examining resilience and coping and comparing the potential mechanisms of resilience and coping in different contexts are highly beneficial.

Fourth, this study did not examine potential moderators of fatigue or coping, such as gender, flight experience, or type of flight operation (i.e., short- or long-haul), which could independently influence both fatigue and coping. Future research should take longitudinal and mixed-methods research approaches, examine different pilot populations, and collect both subjective data (e.g., survey items) and objective data (e.g., observations), which could provide a more comprehensive picture of psychological resilience in the aviation context.

Finally, a potential selection bias cannot be ruled out. There may be systematic differences between pilots who chose to participate in the study and those who did not (e.g., participants may make healthier choices, be more psychologically flexible, or be more confident in their mental health status). Self-selection bias could lead to underrepresentation of individuals experiencing higher distress or lower resilience, which could weaken the relationships detected in our analysis.

## 5. Conclusions

This research underscores the significance of resilience, coping mechanisms, and fatigue as salient psychosocial processes that shape pilots’ mental health and operational safety. Resilience serves as both a primary line of defense that protects against depression, anxiety, and stress, and as secondary line of defense, acting as a moderator of occupational stress through adaptive coping. Fatigue, on the other hand, is a common risk factor that has implications for undermining psychological health and reducing the ability to regulate one’s own emotions. Overall, the findings are a contribution to the growing body of literature that suggests resilience is not so much a personality feature, but a response that can be influenced and nurtured through engagement within environmental and organizational factors.

At the individual level, an avenue of exploration would be the testing of domain-focused resilience building, pilot-specific interventions (i.e., simulation-based stress inoculation, cognitive behavioral coping modalities, mindfulness-based attention training) and skills supplementation as part of the recurrent training for pilots. This may promote additional resiliency and cognitive skills that enable emotional regulation, situational awareness, and decision-making in the context of workplace stress, which are critical contributors to flight safety.

At the organizational level, airlines and aviation authorities should embed psychological resilience and fatigue management within safety management systems (SMS). This may include data-informed fatigue monitoring, stigma-free peer-supporting programs, and structured debriefing practices (or policies) that promote help-seeking behavior with the intention of reaching collective efficacy across flight crews. Such practices reinforce not only the message that the mental health of pilots is not an individual issue but also that collective risk ought to be addressed at the level of aviation safety policy (ICAO, EASA).

From a theoretical perspective, this study advances the integration of the Job Demand–Resource model with the transactional model of stress and coping, offering a process-oriented understanding of how psychological resources interact with occupational stressors to predict mental health outcomes. By situating resilience and coping within this dual-framework model, the study moves the field beyond prevalence-based research toward a mechanistic understanding of psychological adaptation in high-risk professions.

In conclusion, cross-national and longitudinal studies are encouraged to examine the cultural and regulatory contexts of these psychosocial dynamics. Comparative studies across EASA, FAA, and emerging Asian aviation contexts may provide insight into whether resilience manifests differently depending on organizational and cultural factors. In this way, future research can build on current findings to support evidence-based training, engaged safety practices, and coordinated global aviation mental health policies.

## Figures and Tables

**Table 1:** Demographic and Professional Characteristics of Participants (*N* = 200).

Characteristic	*n*	*%*	M	SD
Age				
21–30 years	53	26.5		
31–40 years	103	51.5		
41–50 years	44	22.0		
Gender				
Male	170	85.0		
Female	30	15.0		
Marital Status				
Single	22	11.0		
Married	166	83.0		
Divorced/Widowed/Separated	12	6.0		
Education Level				
Bachelor’s or lower	70	35.0		
Master’s	108	54.0		
PhD	22	11.0		
Flight Experience				
2–10 years	105	52.5		
11–20 years	80	40.0		
21+ years	15	7.5		
Type of Flights				
Domestic	50	25.0		
International	36	18.0		
Both (domestic and international)	114	57.0		
Monthly Flight Hours				
<60 h	110	55.0		
≥61 h	90	45.0		
Sleep Duration (hours/night)			7.77	0.67
Number of Children			2.28	0.72

Note. For categorical variables, values are presented as counts (*N*) and percentages (%). For continuous variables (Sleep Duration, Number of Children), values are presented as the mean (*M*) and standard deviation (*SD*). “Both” refers to pilots who operate on mixed domestic and international routes. Percentages may not total exactly 100% because of rounding. Sleep Duration represents self-reported average nightly sleep hours.

**Table 2:** Descriptive Statistics and Inter-Correlations Among Key Variables (*N* = 200).

Variable	M	SD	Min	Max	Skewness	Kurtosis	1	2	3	4	5	6	7
1. Resilience (CD-RISC)	68.2	9.4	45	95	−0.35	−0.42	—						
2. Depression (DASS-21)	8.5	5.3	0	21	1.02	0.88	−0.052 ***	—					
3. Anxiety (DASS-21)	7.9	5.0	0	21	1.10	1.15	−0.047 ***	0.64 ***	—				
4. Stress (DASS-21)	10.2	5.9	1	21	0.85	0.45	−0.50 ***	0.71 ***	0.68 ***	—			
5. Fatigue Severity (FSS)	30.1	7.2	10	49	−0.18	−0.65	−0.45 ***	0.59 ***	0.60 ***	0.65 ***	—		
6. Task-focused Coping (CISS)	42.4	6.6	26	58	−0.25	−0.10	0.38 ***	−0.21 **	−0.25 **	−0.20 **	−0.28 **	—	
7. Avoidance Coping (CISS)	27.8	5.7	14	42	0.40	0.05	−0.31 **	0.33 **	0.30 **	0.29 **	0.36 ***	−0.22 **	—

Note. *N* = 200. *M* = Mean; *SD* = Standard Deviation. Fatigue was measured using the Fatigue Severity Scale (FSS); coping strategies were assessed with the Coping Inventory for Stressful Situations (CISS); and depression, anxiety, and stress were measured using the DASS-21. Skewness and kurtosis values within ±2 indicate acceptable normality (Tabachnick et al., 2007). All correlations are two-tailed. ** *p* < 0.01. *** *p* < 0.001.

**Table 3:** Hierarchical Multiple Regression Predicting Depression, Anxiety, and Stress.

Outcome	Predictor	β	*ΔR* ^2^	Final *R*^2^
Depression	Resilience	−0.39	0.21	0.35
	Fatigue	0.31	0.09	
	Avoidance coping	0.19	0.05	
Anxiety	Resilience	−0.35	0.18	0.32
	Fatigue	0.34	0.10	
	Avoidance coping	0.12	0.04	
Stress	Resilience	−0.41	0.23	0.36
	Task-focused coping	−0.13	0.07	
	Fatigue	0.29	0.05	
	Avoidance coping	0.18	0.04	

Note. Work-related variables (years of experience, monthly flight hours, and flight type) were entered at Step 1, followed by resilience, fatigue, and coping strategies in subsequent steps. Values are standardized beta coefficients (β). *ΔR*^2^ represents incremental variance explained at each step; final model *R*^2^ values are reported for each outcome (Depression = 0.35; Anxiety = 0.32; Stress = 0.36). *N* = 200. All models significant at *p* < 0.001.

**Table 4:** Moderation of the Stress–Depression Relationship by Resilience.

Predictor	β	SE	T	*p*
Stress	0.42	0.09	4.67	<0.001
Resilience	−0.35	0.08	−4.37	<0.001
Stress × Resilience	−0.18	0.06	−2.99	0.003

Note. PROCESS Model 1, predictors mean-centred. *N* = 200 (subset with complete data). Figure 1 illustrates the interaction (simple slopes at ±1 *SD* resilience).

**Table 5:** Parallel Mediation of the Resilience–Depression Link via Coping Styles.

Path	β	SE	95% CI [LL, UL]
Resilience → Task coping (*a*_1_)	0.38	0.07	[0.24, 0.51]
Resilience → Avoidance (*a*_2_)	−0.31	0.06	[−0.43, −0.19]
Task coping → Depression (*b*_1_)	−0.21	0.08	[−0.36, −0.07]
Avoidance → Depression (*b*_2_)	0.24	0.09	[0.08, 0.41]
Indirect effect *a*_1_*b*_1_	−0.08	0.03	[−0.15, −0.02]
Indirect effect *a*_2_*b*_2_	−0.07	0.03	[−0.13, −0.01]
Total indirect effect	−0.15	0.04	[−0.22, −0.08]

Note. PROCESS Model 4 with 10,000 bootstrap samples. Task-focused and avoidance coping specified as parallel mediators. *N* = 200.

**Table 6:** Depression Scores across Resilience Groups (Low, Medium, High).

Resilience Group	N	*M*	*SD*
Low	103	4.03	4.16
Medium	32	5.66	4.08
High	65	1.31	2.13

Note. One-way ANOVA: *F*(2, 197) = 18.68, *p* < 0.001, *η^2^* = 0.16. Tertiles were created from the continuous resilience score; unequal cell sizes reflect ties at cut-points. Tukey post hoc tests indicated that the high-resilience group reported significantly lower depression than both the low- and medium-resilience groups (both *p* < 0.001), while the low vs. medium comparison was nonsignificant (*p* = 0.07).

## Data Availability

The data that support the findings of this study are available from the corresponding author upon reasonable request.

## Abbreviations

The following abbreviations are used in this manuscript:

CD-RISC	Connor–Davidson Resilience Scale
CISS	Coping Inventory for Stressful Situations
CRM	Crew Resource Management
DAS	depression, anxiety, and stress
DASS-21	Depression Anxiety Stress Scale
EASA	European Aviation Safety Agency
FAA	Federal Aviation Administration
FRMS	Fatigue Risk Management Systems
FSS	Fatigue Severity Scale
JD-R model	Job Demand–Resource model
SMS	safety management systems
